# Incidence and Predictors of Ventricular Arrhythmias in Transthyretin Amyloid Cardiomyopathy

**DOI:** 10.3390/jcm12144624

**Published:** 2023-07-11

**Authors:** Katharina Knoll, Patrick Fuchs, Isabel Weidmann, Fatih Altunkas, Stephanie Voss, Carsten Lennerz, Christof Kolb, Thorsten Kessler, Heribert Schunkert, Wibke Reinhard, Stefan Groß, Teresa Trenkwalder

**Affiliations:** 1 German Heart Centre Munich, Department of Cardiology, Technical University of Munich, 80333 Munich, Germany; 2 DZHK (German Centre for Cardiovascular Research), Partner Site Munich Heart Alliance, 80336 Munich, Germany; 3 German Heart Centre Munich, Department of Cardiovascular Surgery, Technical University of Munich, 80636 Munich, Germany; 4 DZHK (German Centre for Cardiovascular Research), Partner Site Greifswald, 17475 Greifswald, Germany; 5 Department of Internal Medicine B, University Medicine Greifswald, 17475 Greifswald, Germany

**Keywords:** amyloidosis, ventricular tachycardia, ventricular arrhythmia, cardiomyopathy, transthyretin

## Abstract

Background: Wild-type transthyretin amyloid cardiomyopathy (wtATTR-CM) is characterized by heart failure, conduction abnormalities and arrhythmias. The incidence of ventricular arrhythmias, particularly ventricular tachycardias (VTs), in wtATTR-CM is unclear. With the development of targeted therapies and improved overall prognosis, there is an unmet need to identify patients at high risk for VTs who might benefit from ICD therapy. Methods: Between 2017 and 2022, 72 patients diagnosed with wtATTR-CM were prospectively evaluated for the presence of ventricular arrhythmias using a Holter ECG. VTs were defined as >3 consecutive beats with a heart rate > 100 beats per minute originating from a ventricle. Results: The incidence of VTs was 44% (n = 32/72) in unselected wtATTR-CM patients. Patients with VT showed significantly more severe left ventricular (LV) hypertrophy (septum diameter 21 ± 2.6 vs. 19 ± 3.0 mm, *p* = 0.006), reduced LV ejection fraction (47 ± 8 vs. 52 ± 8%, *p* = 0.014) and larger left atria (32 ± 7 vs. 28 ± 6 mm^2^, *p* = 0.020), but no differences in cardiac markers such as NTproBNP and troponin. In a multivariable model, LV hypertrophy (LV mass indexed, OR = 1.02 [1.00–1.03], *p* = 0.031), LV end-diastolic diameter (OR = 0.85 [0.74–0.98], *p* = 0.021) and LV end-systolic diameter (OR = 1.19 [1.03–1.349], *p* = 0.092) were predictive for VT occurrence with an area under the receiver operating characteristic of 0.76 [0.65–0.87]. Conclusions: The incidence of ventricular arrhythmia in wtATTR-CM is high and is associated with an advanced stage of left ventricular disease. Further studies are needed evaluating the role of VTs in predicting sudden cardiac death and the benefit of ICD therapy in wtATTR-CM.

## 1. Background

Transthyretin amyloid cardiomyopathy (ATTR-CM) is an infiltrative myocardial disease. Either pathogenic variants in the transthyretin gene in hereditary ATTR-CM (hATTR-CM) or still unknown mechanisms in senile or wild-type ATTR-CM (wtATTR-CM) cause an extracellular deposition of misfolded transthyretin protein in the heart [1]. Due to advances in diagnostic strategies, imaging modalities and increased awareness of the disease, ATTR-CM has been more commonly diagnosed over the past 10 years. Indeed, the condition is now thought to be a leading cause of heart failure, affecting up to 13% of patients with heart failure with preserved ejection fraction (HFpEF) aged over 60 years [2].

The cardinal symptoms of ATTR-CM are progressive heart failure, conduction disorders and arrhythmias, affecting both atria and ventricles [1]. Compared with light-chain amyloid cardiomyopathy (AL-CM), ATTR-CM shows a higher prevalence of atrial arrhythmias and atrioventricular conduction disease, with a four- to sixfold higher prevalence of atrial fibrillation in a retrospective analysis [3]. Ventricular arrhythmias, in contrast, are commonly found in light chain amyloidosis, affecting up to 65% of patients in a stem cell transplant unit monitored by telemetry over a median of 24 days [4]. Analyzing specifically non-sustained ventricular tachycardias (nsVT), these were detected in 18% of unselected patients with AL-CM on 24 h Holter monitoring [5]. In hereditary transthyretin amyloidosis with a ATTR Val30Met mutation, an observational study revealed a similar incidence of 17% for ventricular tachycardias (VT) on 24 h Holter monitoring [6]. Data regarding the occurrence of ventricular arrhythmias and especially VTs in wtATTR-CM are currently scarce.

Even more limited is the evidence regarding the use of implantable cardioverter defibrillators (ICDs) in ATTR-CM [1]. Observational studies revealed no benefit of primary prevention ICDs in patients with ATTR-CM [7,8,9,10,11]. However, these studies have several limitations, such as a retrospective character, small sample size and heterogeneous patient groups not differentiating AL- and ATTR amyloid subtypes [7,8,9,10].

Besides the limited evidence of a clinical benefit, pathophysiological considerations suggest limited value of ICD therapy in amyloidosis. Historically, the main cause of death in this patient population has been progressive heart failure, leading to electromechanical dissociation and pulseless electric activity rather than ventricular arrhythmias [12], and is not influenced by the presence of an ICD [8]. Based on limited data, contemporary guidelines (ESC, HRS) recommend ICDs for secondary prevention with a class IIa evidence level C recommendation [13], while no recommendation for primary prevention is expressed [1,2,13,14].

Due to increased disease awareness [15] and earlier diagnosis (at younger ages and less advanced stages), possibly prompted by new targeted therapies improving prognosis [16], the role of ICD therapy for ATTR-CM is undergoing a re-evaluation [14]. However, there is a lack of predictive parameters for occurrence of sudden cardiac death or benefit from ICD in this patient group. Previous studies suggested VTs being of value in patients with cardiac amyloidosis for predicting disease progression [17], appropriate ICD therapy [18], or mortality [10,19,20]. Indeed, presence of any clinically apparent arrhythmia during hospitalization in patients with amyloid cardiomyopathy has been associated with worse prognosis [21]. Therefore, some authors suggested ICD implantation in case of unexplained syncope or occurrence of nsVT and expected survival over 1 year (Stanford Amyloid Center’s ICD implantation criteria) [20].

Hence, there is a need to identify patients who are at high risk for or suffer from ventricular arrhythmias who might possibly benefit from targeted therapy. Therefore, we prospectively analyzed the incidence and clinical predictors of ventricular arrhythmias in an all-comers population with proven diagnosis of wtATTR-CM.

## 2. Methods

Between 2017 and 2022, we prospectively investigated the presence of ventricular arrhythmias in consecutive patients diagnosed with wtATTR-CM at a tertiary referral center in Germany. The study was approved by the local ethics committee of the Technical University of Munich and was conducted according to the principles of the Declaration of Helsinki. The study was registered at German Clinical Trial Register with the identifier DRKS00031433. All patients provided written informed consent to participate in the study.

### 2.1. Study Cohort

All patients included in this study had a confirmed diagnosis of wtATTR-CM. Diagnosis of ATTR-CM was confirmed either by detection of ATTR amyloid deposits in endomyocardial biopsy or according to a non-invasive algorithm with DPD-bone scintigraphy and laboratory assessment for (exclusion of) monoclonal proteins [1,14,22]. In addition, all patients with ATTR-CM underwent genetic sequencing of the transthyretin gene and only patients without a genetic mutation in the transthyretin gene were included in this analysis.

### 2.2. Assessment of Ventricular Arrhythmias

Prevalence and severity of arrhythmias were assessed through ambulatory 24 h Holter ECG monitoring with a 3- or 12-lead electrocardiogram (Schiller) by nurses and physicians specifically trained for the presence of ventricular arrhythmias. Ventricular arrhythmias were defined according to the 2022 ESC guidelines for the management of patients with ventricular arrhythmias and the prevention of sudden cardiac death [13] and included the following: premature ventricular complex (PVC), couplets (2 consecutive PVCs), triplets (3 consecutive PVCs), ventricular tachycardias (VTs, >3 consecutive beats with a heart rate > 100 beats per minute (b.p.m.) originating from the ventricle), ventricular fibrillation and accelerated idioventricular rhythm (IVR, ectopic ventricular rhythm with rate between 60 and 100 b.p.m. at rest [23]). VTs were then further characterized as non-sustained ventricular tachycardia (nsVT, VT persisting up to less than 30 s) and sustained ventricular tachycardia (sVT, VT persisting more than 30 s or requiring intervention for termination). Moreover, we evaluated the presence of conduction disorders (including atrioventricular block, left and right bundle branch block, hemiblock) as well as atrial arrhythmias (including atrial fibrillation).

### 2.3. ECG, Echocardiography and Laboratory Analyses

In addition to Holter ECG monitoring, all patients underwent a comprehensive clinical evaluation including assessment of comorbidities, medication and current NYHA class. All echocardiographic studies were performed by experienced institutional cardiologists and echocardiographic measures were assessed according to the current guideline recommendations [24] using the Philipps EPIQ CVx. Systolic function was measured using the biplane Simpson’s method of disks. The left ventricular mass was calculated using the Devereux formula [25] based on 2D-guided values obtained in the parasternal long axis and then divided by the body surface area to obtained the indexed left ventricular mass. Left ventricular diastolic function was assessed and graded according to current guidelines [26], using tissue Doppler velocities of the septal and lateral mitral valve annulus and pulse-wave Doppler profiles across the mitral valve leaflets. Right ventricular function was assessed through measurement of the tricuspid annular plane systolic excursion (TAPSE) in the lateral tricuspid annulus from the apical 4-chamber view using an M-mode cursor.

Besides conventional echocardiographic parameters, we also assessed the global longitudinal strain using the AutoStrain LV (Philipps) analysis.

In addition, laboratory parameters were collected including NTproBNP, troponin T, renal function (GFR), electrolytes (sodium, potassium), hemoglobin, and platelet and white blood cell counts.

### 2.4. Statistical Analysis

Data are presented as mean ± SD or median (interquartile range) for continuous variables, as appropriate, and as frequencies (percentages) for categorical variables. For baseline variables, pairwise comparisons were made with the non-parametric Mann–Whitney U test for continuous variables, and with the Pearson χ^2^ test for categorical variables. In order to identify predictors for VT occurrence, we performed two separate multivariable analyses, focusing on clinical as well as laboratory values in the first model and echocardiographic parameters in the second model. In both models, variables were selected for clinical relevance and avoiding combinations that would lead to collinearity. In both models, we used a conservative threshold of *p* = 0.157 for retaining variables in the model by a stepwise backward-selection procedure. This threshold is equivalent to the AIC criterion for selection of predictors [27]. Finally, we combined the retained variables identified in the first two models in a third comprehensive model and used the same backward selection procedure again. Statistical analyses were performed with Stata 17.1 (StataCorp LLC., College Station, TX, USA), with *p*-values < 0.05 considered statistically significant.

## 3. Results

Between 2017 and 2022, 77 patients were enrolled in this registry and prospectively assessed by Holter ECG for the occurrence of ventricular arrhythmias. Of those, five patients were excluded from the final analysis due to either missing clinical data or technical defects of the remote ECG monitoring. Finally, 72 patients were analyzed with a mean duration of 36.5 h of Holter ECG (maximum duration 7 days, minimum 12 h, total duration 2559 h). The corresponding baseline characteristics are shown in Table 1 and Table 2.

### 3.1. Prevalence of Arrhythmias on Holter

The mean heart rate during the Holter monitoring was 69 ± 11 b.p.m. Almost all patients presented with single PVCs (98% (n = 69/70)), 86% (n = 59/69) with couplets, 56% (n = 41/70) with triplets, 46% (n = 31/68) with one or more episodes of bigeminy, 41% (n = 28/68) with trigemini, 29% (n = 20/68) with IVR and 44% (n = 32/72) with at least one VT. Of those patients presenting with VTs, all presented with self-terminating nsVTs and none with sVTs. PVCs accounted from <0.01% up to 13.5% of all heart beats (median 0.6%). Bradyarrhythmias were also common, with 9% (n = 6/68) of patients showing intermittent AV-block and 41% (n = 29/71) presenting with at least one pause over 2 s, the longest with a duration of 3.6 s. Atrial arrhythmias were also detected frequently with 51% (n = 37/72) of patients presenting with atrial fibrillation.

### 3.2. Predictors of Ventricular Tachycardias

Comparing baseline parameters of patients presenting with and without VTs, we found significant differences in several echocardiographic parameters (Table 2). Patients with VTs presented more severe left ventricular hypertrophy, as reflected by significantly larger septal diameter (IVSD) and posterior left ventricular wall diameter (PWLVD) as well as a trend towards increased left ventricular mass indexed for body surface area (LVM). Furthermore, wtATTR-CM patients with VTs showed significantly larger left atria and significantly reduced ejection fraction (LVEF) compared to those without VTs. In contrast, no statistically relevant differences were found in heart failure medication and comorbidities, laboratory parameters, as well as most ECG variables. Patients with VTs were more frequently treated with tafamidis during the Holter assessment.

We evaluated the multivariable predictive performance of different clinical and echocardiographic variables for discriminating between patients with and without VTs (Table 3), separately in models assessing either clinical or echocardiographic parameters through a stepwise backward-selection procedure using a conservative threshold *p*  =  0.157 equivalent to the AIC criterion for retaining variables in the models.

**Figure 1:**
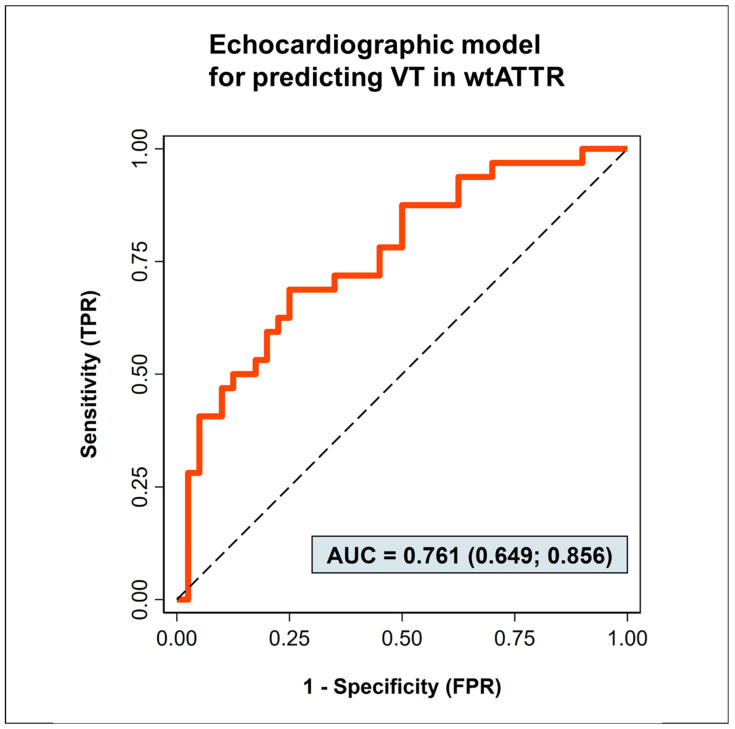
Echocardiographic model for occurrence of ventricular tachycardias in patients with wtATTR-CMP.

Clinical parameters, specifically GFR (OR = 1.03 [0.99–1.06], *p* = 0.073) and troponin T (OR = 1.02 [0.99–1.04], *p* = 0.058), showed only trends for predicting the occurrence of VTs in the first model (AUC 0.62, see Supplementary Figure S1). Echo-parameters, including left ventricular mass per body mass index (LVM, OR = 1.02 [1.00–1.03], *p* = 0.031), left ventricular end-diastolic diameter (LVEDD, OR = 0.85 [0.74–0.98], *p* = 0.021), and left ventricular end-systolic diameter (LVESD, OR = 1.19 [1.03–1.349], *p* = 0.092) were retained in the second model that produced an area under the receiver operating characteristic (AUROC) of 0.76 [0.65–0.87] as shown in Figure 1. In a third model combining both previously retained clinical and echocardiographic parameters, only the echocardiographic variables were retained after a repeated backward selection, resulting in the same AUROC as the sole echocardiographic model (see Supplementary Figure S2).

## 4. Discussion

In this prospective study on the prevalence of ventricular arrhythmias in wtATTR-CM, 98% of patients presented with PVC and 44% with VTs. Severe ventricular arrhythmias were rare, and no sVT were detected through ambulatory Holter monitoring over 107 days and 7 h. NsVTs, on the other hand, were commonly found with a prevalence of 44% in this unselected cohort.

This finding is of importance because there is a lack of data regarding the prevalence and relevance of nsVTs in wtATTR-CMP. VTs have been considered important risk factors for worse outcomes and increased mortality in patients with cardiac amyloidosis in previous studies [10,19,20]. However, the evidence regarding prevalence and relevance of nsVT is still limited. Moreover, the current publications analyzed a mixed cohort of cardiac amyloidosis patients without further differentiation of specific subtypes (AL, hATTR and wtATTR-patients), while there is a lack of data regarding the clinically most common subgroup of wtATTR-CM patients.

A second finding of our study is the characterization of wtATTR-CM patients with arrhythmias. In our cohort, patients with VT presented with worse left ventricular ejection fraction and more severe left ventricular hypertrophy when compared with patients without VT. Moreover, an increased left ventricular end-systolic diameter, an indirect marker for reduced ventricular contraction as reflected in the Teichholz formula, was predictive of VT occurrence in the multivariate predictive model.

These observations regarding the EF and LVESD are consistent with observational studies in ATTR-CM ICD carriers, where ventricular arrhythmias were significantly more common in patients with an EF below 40% compared to an EF above 40% [9]. Moreover, the association between impaired EF and ventricular arrhythmias is analogous to other cardiomyopathies. For example, the indication for primary prevention ICD in ischemic cardiomyopathy is mainly based on a reduced ejection fraction below 35% [13]. In addition, a reduction in EF below 50% is an additional risk factor for arrhythmias in patients with hypertrophic cardiomyopathy [13]. However, in ATTR-CM, it might be detrimental to solely focus on systolic ejection fraction. Due to the infiltrative pathophysiology of cardiac amyloidosis, first leading to a diastolic/restrictive cardiomyopathy and only in advanced stages to an impairment of systolic function [28], the reduced ejection fraction might be an accurate but late marker for ventricular arrhythmias or even for worse prognosis [29]. Consequently, patients with ATTR-CM and impaired ejection fraction might possibly not benefit from ICD therapy. Despite experiencing ventricular arrhythmias, those patients might suffer from severe and progressive heart failure, leading to acute cardiac failure and electromechanical dissociation rather than arrhythmias as main causes of death. Thus, the identification of new prognostic markers besides ejection fraction is of utmost importance.

The second factor associated with presence of VT and predictive for VT occurrence in our observational study was left ventricular hypertrophy, quantified as left ventricular mass per body mass index using the Devereux formula [25]. Assessment of left ventricular hypertrophy by measurement of left ventricular mass or intraventricular septum diameter is recommended to detect and quantify disease progression in ATTR-CM [28,30]. Similarly, in regard to left ventricular ejection fraction, left ventricular mass is only a late marker of disease, as it increases in advanced disease stages [28].

Our study suggests a prognostic value of both left ventricular function and left ventricular mass for VT occurrence. Further studies will be needed to establish a cutoff value to identify patients at higher risk for ventricular arrhythmias that might benefit from closer rhythm monitoring or even therapeutic intervention. Preferably, these values could be added to a risk score identifying patients that benefit from ICD therapy.

Other echocardiographic markers, such as reduction in global longitudinal strain or impaired diastolic function, both earlier markers of ATTR-CM, were not significantly different in patients with VT and had no predictive value of VT occurrence. Likewise, NTproBNP, a traditional marker for heart failure, did not predict occurrence of ventricular tachycardia in our cohort. Only troponin and GFR showed trends for predicting VT occurrence but were not retained in a model that also included echocardiographic parameters. This is of particular importance, as both NTproBNP, troponin and GFR have been proposed as prognostic markers for overall survival in ATTR-CM [28,31,32].

Our data, which are in line with those of previous publications [20], suggest those biomarkers are of limited prognostic value for predicting occurrence of arrhythmias. While troponin might have some value in this context by reflecting myocardial damage, NTproBNP might be more reflective of heart failure and volume overload than of arrhythmias.

Besides echocardiographic and laboratory markers, the role of comorbidities was also assessed. In this setting, the assessment of concomitant coronary artery disease was of particular interest. In clinical practice, the diagnosis of VT often triggers a workup to rule out cardiac ischemia [13]. In this study, presence of coronary artery disease was not associated with VT occurrence, suggesting the cardiomyopathy being the primary cause of arrhythmias. Nevertheless, in first occurrence of non-sustained VT or in case of hemodynamically relevant VT or concomitant ischemic symptoms, progression or new onset of coronary artery disease should be excluded.

Similar to comorbidities, patients with and without VT showed no significant differences in baseline heart failure medications in our observational study. In this context, betablockers are of particular interest due to their antiarrhythmic properties, which might suppress or reduce occurrence of arrhythmias, both atrial and ventricular. On the other hand, betablockers are often poorly tolerated in ATTR-CM due to its restrictive pathophysiology with preserved ejection fraction and reduced stroke volumes. The finding of VT in ATTR-CM with hypotension or concomitant conduction delays often imposes a therapeutic challenge, balancing benefits of betablocker therapy against possible harm. Another potent antiarrhythmic drug for treatment of ventricular arrhythmias is amiodarone. Given its unfavorable side effects, it is rarely administered chronically. In our study, only one patient was on amiodarone treatment at baseline, and none was administered amiodarone as a consequence of the Holter results.

Besides heart failure and antiarrhythmic treatment, we also assessed amyloidosis-specific treatment. Currently, the only drug approved for treatment of wtATTR-CM is tafamidis, a transthyretin stabilizer [1]. Tafamidis is a benzoxazole derivative that binds the thyroxine-binding sites of transthyretin, thus stabilizing transthyretin tetramers, inhibiting the dissociation into monomers [16]. Tafamidis has been shown to slow disease progression and improve prognosis in wtATTR-CM compared to placebo [16]. In our exploratory study analyzing occurrence of arrhythmias on Holter monitoring, only 16% (n = 12/72) of patients were on tafamidis therapy during the Holter assessment. This number substantially increased at a follow-up assessment, when two-thirds of patients (68%, n = 49/72) were either recommended or prescribed tafamidis.

Interestingly, wtATTR-CM patients presenting with VTs were significantly more commonly on tafamidis compared to patients without VTs during Holter. However, this changed at follow-up, when the prescription pattern was similar in both groups. One possible explanation of this finding is a prompt initiation of disease-specific therapy in patients suffering from more severe disease. Supposably, patients with more prominent left ventricular hypertrophy on imaging, and more severe clinical symptoms, may be more easily diagnosed, and in consequence receive earlier treatment. Furthermore, some patients were referred to our tertiary center for evaluation of a disease-specific therapy and thus were not yet receiving tafamidis treatment during Holter monitoring. However, as this study is of explorative nature, a definitive assessment of this finding is not possible. Further studies to assess the effect of treatment, both antiarrhythmic (medical and ICD) as well as disease-specific, on occurrence and clinical effect of VTs are needed.

## 5. Strengths and Limitations

The strength of this study is its focus on wtATTR-CM in a phenotypically well-characterized cohort. To our knowledge, this is the first prospective study on wtATTR-CM analyzing the prevalence and predictors of ventricular arrhythmias in wtATTR-CM with a systematic approach using Holter ECG. Furthermore, our use of Holter ECG monitoring reflects the everyday clinical practice and is thus highly generalizable. Although more continuous ECG monitoring, such as implantation of event recorders or continuous rhythm monitoring on hospital ward, might reveal even higher incidences of ventricular tachycardias, these approaches are difficult to implement.

The main limitation of this study is the cohort size and thus the limited number of events of interest. This limits the ability to use larger comprehensive multivariable models as a starting point for the development of a final prediction model. Therefore, our promising results need replication and validation in larger patient cohorts for robust conclusions. One further important limitation is the currently limited data on patient outcomes. Further studies are needed to validate our results and to assess the effect of VTs detected by Holter on overall survival. In addition, there is a need to analyze whether ICD or antiarrhythmic therapy in patients with advanced stages of wtATTR-CM improves prognosis.

Finally, the data of this study were from patients with and without targeted therapy for ATTR-CM. The effect of such a therapy on the prevalence of ventricular arrhythmias but also on their prognostic implications should be assessed in further studies.

## 6. Conclusions

The incidence of ventricular arrhythmias in an all-comers population of patients with wtATTR-CM was high, affecting approximately half of patients. Patients presenting with ventricular arrhythmias showed an advanced stage of left ventricular disease, with reduced LVEDD, increased LVESD and increased LV mass indexed by BSA being promising predictors of VTs in a multivariable predictive model. Further larger studies validating the predictive value of these variables are needed, including the assessment of VTs in predicting sudden cardiac death, possibly allowing their use in a clinical risk score.

## Figures and Tables

**Table 1:** Baseline clinical characteristics and differences between wtATTR-CM patients with and without ventricular tachycardia on Holter.

	ATTR-CM without Ventricular Tachycardia	ATTR-CM with Ventricular Tachycardia	*p*-Value
	n = 40	n = 32	
**Clinical characteristics**			
Age (years; median, IQR)	81 (76–84)	80 (75–82)	0.385
Gender (male; n, %)	38 (95)	31 (97)	1.000
Body mass index (kg/m^2^; median, IQR)	24.8 (23.3–27.0)	24.6 (23.1–28.1)	0.882
Angina pectoris (n, %)	6 (15)	6 (19)	0.756
Dyspnea (n, %)	32 (80)	30 (94)	0.169
Syncope (n, %)	4 (10)	3 (9)	1.000
Systolic blood pressure (mmHg; median, IQR)	135 (122–148)	140 (125–149)	0.489
**Medication at baseline**			
Beta blocker (n, %)	23 (58)	24 (75)	0.142
ARNI (n, %)	2 (5.0)	2 (6.3)	1.000
ACE-inhibitor or AT-receptor antagonist (n, %)	26 (65)	22 (69)	0.805
Mineralocorticoid receptor-antagonist (n, %)	16 (40)	15 (47)	0.635
SGLT2-antagonist (n, %)	7 (18)	2 (6.3)	0.282
Diuretic (n, %)	28 (70)	26 (81)	0.412
Amiodarone (n, %)	1 (2.5)	0 (0)	1.000
Tafamidis during Holter (n, %)	2 (5)	10 (31)	0.004
Tafamidis at follow-up (n, %)	25 (63)	24 (75)	0.402
**Comorbidities**			
Coronary artery disease (n, %)	23 (58)	19 (59)	0.611
Atrial fibrillation (n, %)	25 (63)	20 (63)	1.000
Pacemaker (n, %)	6 (15)	2 (6.3)	0.287
Implantable cardioverter defibrillator (ICD, n, %)	2 (5.0)	1 (3.1)	1.000
Cardiac resynchronization therapy (CRT, n, %)	1 (2.5)	1 (3.1)	1.000
**Laboratory values**			
NTproBNP (pg/mL; median, IQR)	2945 (1615–5900)	3100 (1705–4875)	0.825
Troponin T (ng/L; median, IQR)	46 (32–63)	53 (42–70)	0.141
Creatinine (mg/dL; median, IQR)	1.3 (1.03–1.62)	1.1 (1.07–1.32)	0.109
GFR (ml/min; median, IQR)	56 (43–75)	63 (55–74)	0.245
Potassium (K^+^, mmol/L; median, IQR)	4.37 (4.05–4.62)	4.18 (3.96–4.43)	0.124
Sodium (Na^2+^, mmol/L; median, IQR)	139 (137–141)	140 (138–142)	0.082

**Table 2:** Baseline echocardiographic and ECG characteristics and differences between wtATTR-CM patients with and without ventricular tachycardia on Holter.

	ATTR-CM without Ventricular Tachycardia	ATTR-CM with Ventricular Tachycardia	*p*-Value
	n = 40	n = 32	
**Echocardiography** (all: median, IQR)			
Interventricular septal diameter (mm)	19 (17–20)	21 (19–22)	**0.006**
Left ventricular end-diastolic diameter (mm)	43 (39–48)	44 (39–49)	0.972
Left ventricular end-systolic diameter (mm)	31 (27–36)	33 (30–40)	0.063
Left ventricular posterior wall diameter (mm)	15 (13–17)	17 (15–19)	**0.026**
LV mass indexed for body surface area (g/m^2^)	170 (132–199)	198 (175–221)	0.063
Ejection fraction (Simpson, %)	55 (48–57)	47 (41–52)	**0.014**
TAPSE (mm)	13.5 (12–18)	14.0 (10–15)	0.470
Left atrial area (mm^2^)	28 (24–32)	31 (27–36)	**0.020**
Right atrial area (mm^2^)	24 (21–29)	24 (22–39)	0.430
E/e’	18.3 (13.8–21.5)	17.7 (13.6–23.1)	0.773
GLS	−9.3 (−11.4–−7.9)	−8.4 (−11.5–−6.1)	0.533
**Electrocardiogram**			
QRS-complex duration (ms; median, IQR)	118 (103–140)	116 (103–140)	0.830
QTc-interval duration (ms; median, IQR)	481 (462–507)	489 (468–509)	0.325
T wave inversion (n, %)	9 (23)	5 (15)	0.553
LBBB (n, %)	2 (5)	1 (3)	1.000
RBBB (n, %)	5 (13)	5 (16)	0.746
LAHB (n, %)	12 (31)	17 (53)	0.089
LPHB (n, %)	6 (15)	0 (0)	**0.029**
Low-voltage pattern (n, %)	13 (33)	7 (21)	0.375

**Table 3:** Variables included in the initial and final multivariable predictive models.

Variables Included in the Multivariable Model	*p*-Value	Odds Ratio [95% Confidence Interval]
**Clinical parameters**		
Age	0.858	-
BMI	0.499	-
Coronary artery disease	0.814	-
Dyspnoea (NYHA)	0.732	-
Syncope	0.651	-
NTproBNP (pg/mL)	0.778	-
Troponin T (ng/L)	0.058	1.02 [0.999–1.05]
GFR (ml/min)	0.073	1.03 [0.997–1.06]
Systolic blood pressure	0.765	-
**Echocardiographic parameters**		
Interventricular septal diameter (mm)	0.763	-
Left ventricular end-diastolic diameter (mm)	**0.021**	0.85 [0.74–0.98]
Left ventricular end-systolic diameter (mm)	**0.022**	1.19 [1.03–1.39]
Left ventricular posterior wall diameter (mm)	0.591	-
LV mass indexed for body surface area (g/m^2^)	**0.031**	1.02 [1.001–1.03]
Ejection fraction (Simpson, %)	0.422	-
TAPSE (mm)	0.381	-
Left atrial area (mm^2^)	0.257	-
Right atrial area (mm^2^)	0.553	-
E/e’	0.306	-

## Data Availability

Due to privacy and ethical constrictions, the data are not publicly available but can be obtained from the corresponding author upon reasonable request.

## Supplementary Materials

The following supporting information can be downloaded at: https://www.mdpi.com/article/10.3390/jcm12144624/s1, Figure S1. Second, echocardiographic model for occurrence of ventricular tachycardias in patients with wtATTR-CMP. Figure S2. Comparison of the first and second model for predicting VT occurrence.
