# Supplementary material for: Incidence and Predictors of Ventricular Arrhythmias in Transthyretin Amyloid Cardiomyopathy
Source: J Clin Med. 2023 Jul 11;12(14):4624. doi: 10.3390/jcm12144624 (PMC10380522; doi:10.3390/jcm12144624)
Supplement: Supplementary file 1 [file jcm-12-04624-s001.zip › jcm-2485203-supplementary.pdf]

Supplement

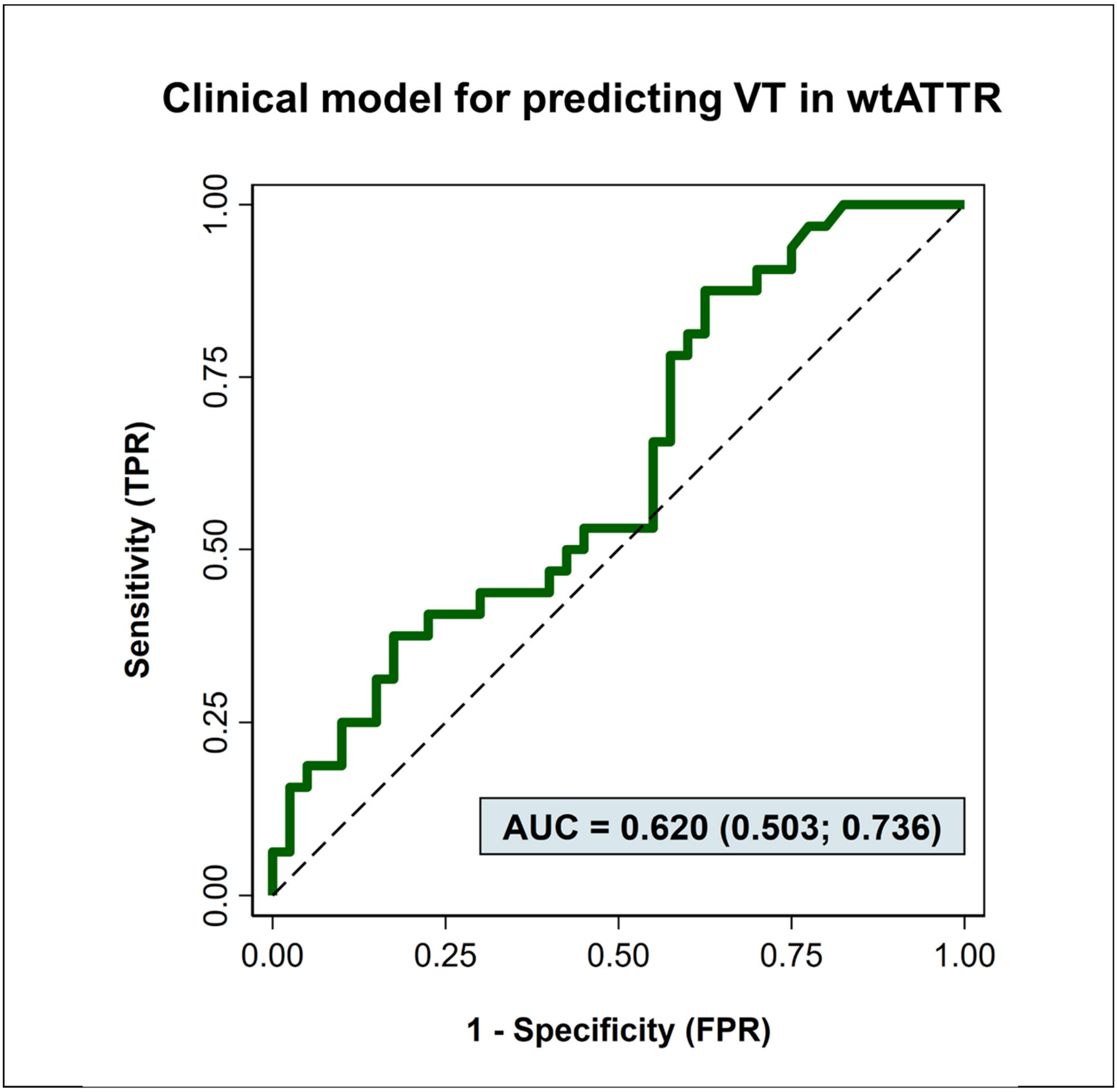

Supplementary Figure S1: Second, echocardiographic model for occurrence of ventricular tachycardias in patients with wtATTR-CMP

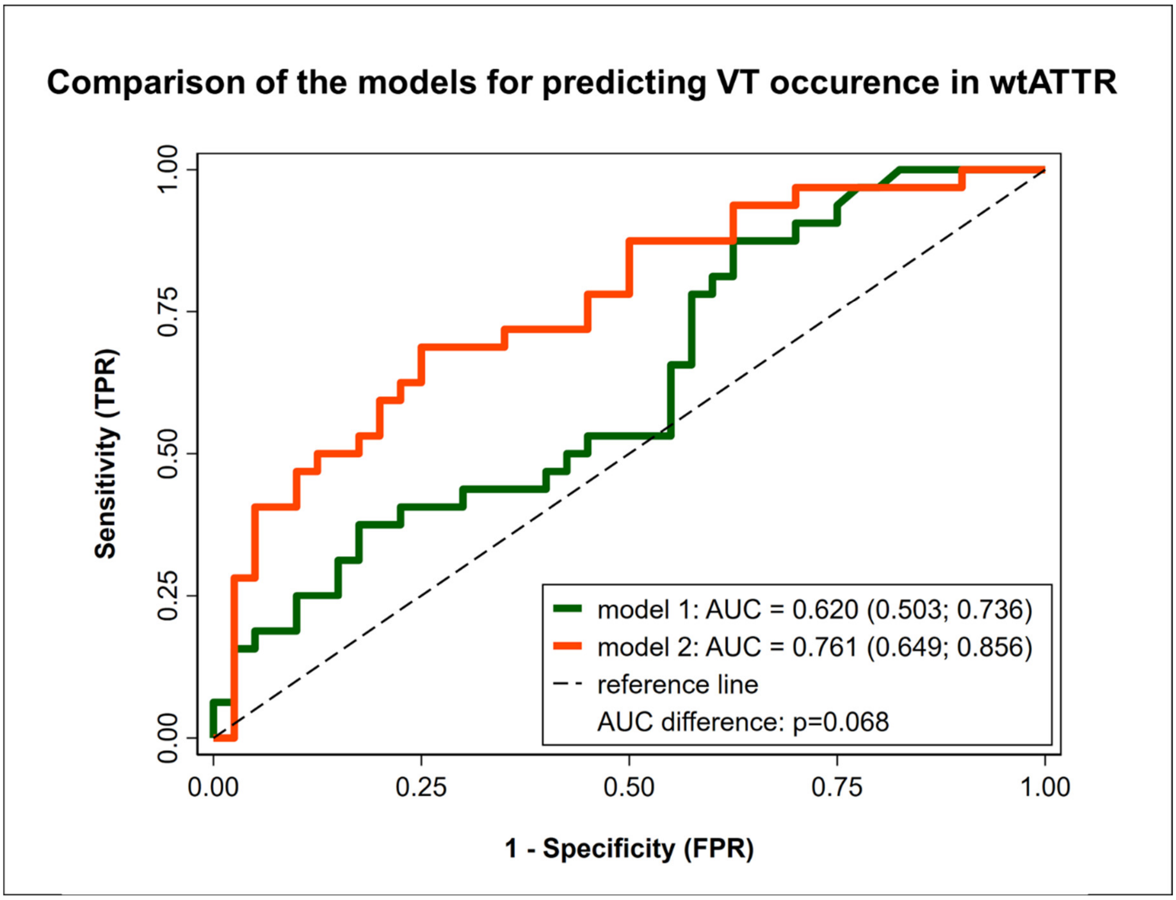

Supplementary Figure S2: Comparison of the first and second model for predicting VT occurrence
